# Associations between thyrotropin receptor antibody, rheumatoid autoantibodies, and disease activity in patients with rheumatoid arthritis: a retrospective cross-sectional study

**DOI:** 10.3389/fmed.2026.1970456

**Published:** 2026-10-21

**Authors:** Hongwei He, Qianqian Wang, Yun Lu, Yongqiang Zhang, Jiang Liang

**Affiliations:** 1 Guizhou University of Traditional Chinese Medicine, Guiyang, Guizhou, China; 2 Qianxinan Prefecture Hospital of Traditional Chinese Medicine, Xingyi, Guizhou, China; 3 The First Affiliated Hospital of Guizhou University of Traditional Chinese Medicine, Guiyang, Guizhou, China

**Keywords:** anti-cyclic citrullinated peptide antibody, autoimmune comorbidity, disease activity, rheumatoid arthritis, rheumatoid factor, thyrotropin receptor antibody

## Abstract

**Objective:**

Thyrotropin receptor antibody (TRAb) frequently coexists with rheumatoid factor (RF) and anti-cyclic citrullinated peptide antibody (anti-CCP) in patients with rheumatoid arthritis (RA), but its association with disease activity remains unclear. This study assessed the associations of TRAb and rheumatoid autoantibodies with DAS28-ESR in RA patients.

**Methods:**

This retrospective cross-sectional study enrolled 192 RA patients hospitalized at the First Affiliated Hospital of Guizhou University of Traditional Chinese Medicine between October 2024 and October 2025, all meeting the 2010 ACR/EULAR criteria. Patients were classified as TRAb-positive or TRAb-negative using the clinical cutoff of 1.75 IU/L. Associations between TRAb and DAS28-ESR were evaluated by Spearman correlation, restricted cubic spline (RCS) analysis, and multivariable linear regression. The primary analysis was repeated in euthyroid patients (*n* = 167). Interactions of TRAb with Ln(RF) and Ln(anti-CCP) were tested in all 192 patients, and an exploratory four-group comparison was performed in 138 patients with concordant antibody titers.

**Results:**

TRAb correlated positively with DAS28-ESR (*ρ*=0.36, *P* < 0.001), and RCS analysis revealed a nonlinear association (*P* = 0.002 for nonlinear). hs-CRP, ESR, and DAS28-ESR were higher in TRAb-positive (*n* = 51) than TRAb-negative patients (*n* = 141; all *P* < 0.001). After covariate adjustment, TRAb positivity remained independently associated with higher DAS28-ESR (*B* = 1.457, *P* < 0.001), and the association persisted in euthyroid patients (4.9 vs. 4.1, *P* < 0.001). TRAb showed a statistically significant interaction with Ln(anti-CCP) (*P* = 0.0014) and a borderline interaction with Ln(RF) (*P* = 0.046); DAS28-ESR was highest in TRAb-positive patients with high-titer autoantibodies.

**Conclusions:**

TRAb positivity was associated with higher disease activity and systemic inflammation in RA patients, including those with normal thyroid function, and the association strengthened with increasing rheumatoid autoantibody titers. The value of combined TRAb, RF, and anti-CCP testing for immune stratification in RA warrants prospective validation.

## 1 Introduction

Rheumatoid arthritis (RA) is a systemic autoimmune disease characterized mainly by chronic synovial inflammation and destruction of articular bone and cartilage (1). There is prominent individual heterogeneity in disease activity among patients. In clinical practice, precise inflammatory stratification is of great significance for guiding clinical treatment and evaluating prognosis (2–4). DAS28-ESR is a widely used tool for assessing RA disease activity in clinical settings. However, this score mainly relies on joint physical signs and acute-phase inflammatory markers, and provide limited insight into the full autoimmune antibody burden in individual patients (5–7). Rheumatoid factor (RF) and anti-cyclic citrullinated peptide (anti-CCP) antibodies are key pathogenic autoantibodies in RA. Their titers are closely correlated with joint damage, treatment response and extra-articular adverse outcomes, making them core biological markers for RA risk stratification (8–10).

Comorbidity is a prominent clinical feature of autoimmune diseases (11), and patients with RA frequently present with comorbid organ-specific autoimmune thyroid diseases (AITDs) (12–14). Graves' disease (GD) is one of the major subtypes of AITDs. TRAb, the characteristic autoantibody of Graves' disease (GD), was previously thought to exert pathogenic effects exclusively in thyroid tissues. Recent studies have suggested that, apart from its action on the thyroid, TRAb may participate in extrathyroidal immunomodulation through TSH-receptor-related signaling pathways (15); however, the current evidence remains insufficient, and this hypothesis awaits direct *in vivo* support. Epidemiological evidence indicates that the positive rate of TRAb is higher in RA patients than in the general population. RA and AITDs share several overlapping genetic susceptibility loci. Polyclonal B-cell activation triggers the concurrent synthesis of various autoantibodies, which explains the coexistence of multiple autoantibodies as well as the comorbidity of RA and AITDs in a single patient (16, 17).

Most prior studies have only examined the bivariate association between TRAb positivity and RA clinical phenotypes (18), while few conduct stratified analyses combined with RF and anti-CCP titers. It remains unclear whether TRAb and rheumatoid autoantibodies merely coexist or each independently contributes to the inflammatory burden. If the coexistence of TRAb positivity and high-titer rheumatoid autoantibodies is associated with a graded increase in disease activity, it would indicate that the association between TRAb and disease activity may be titer-dependent, thereby supplementing the current stratification framework for RA. Therefore, based on a single-center retrospective cross-sectional study, this research analyzes the association between TRAb and rheumatoid autoantibodies and their relationship with DAS28-ESR in RA patients, explores the disease activity patterns when the two types of antibodies coexist, and provides real-world evidence for immune-based stratified management of RA.

## 2 Materials and methods

### 2.1 Research object

This was a single-center retrospective cross-sectional study reported in accordance with the STROBE statement (Supplementary Material S1). A total of 447 hospitalized patients with a diagnosis of rheumatoid arthritis (RA) at the First Affiliated Hospital of Guizhou University of Traditional Chinese Medicine between October 2024 and October 2025 were screened (counted as individual patients; for those with multiple admissions, only the first admission was retained), of whom 192 patients were finally included to constitute the overall study cohort for baseline comparisons, correlation analyses, and regression modeling. The screening process is shown in Figure 1. Exclusion criteria were malignancy (*n* = 22), acute infection or severe multi-organ failure (*n* = 44), missing key clinical or laboratory data (*n* = 169), and repeat admissions (*n* = 20). All included patients had complete TRAb, RF, anti-CCP, hs-CRP, and ESR measurements, a standardized DAS28-ESR disease activity score, and complete baseline clinical data. The DAS28-ESR score was calculated by the attending physician using an online medical calculator, according to the following formula (19):

**Figure 1:**
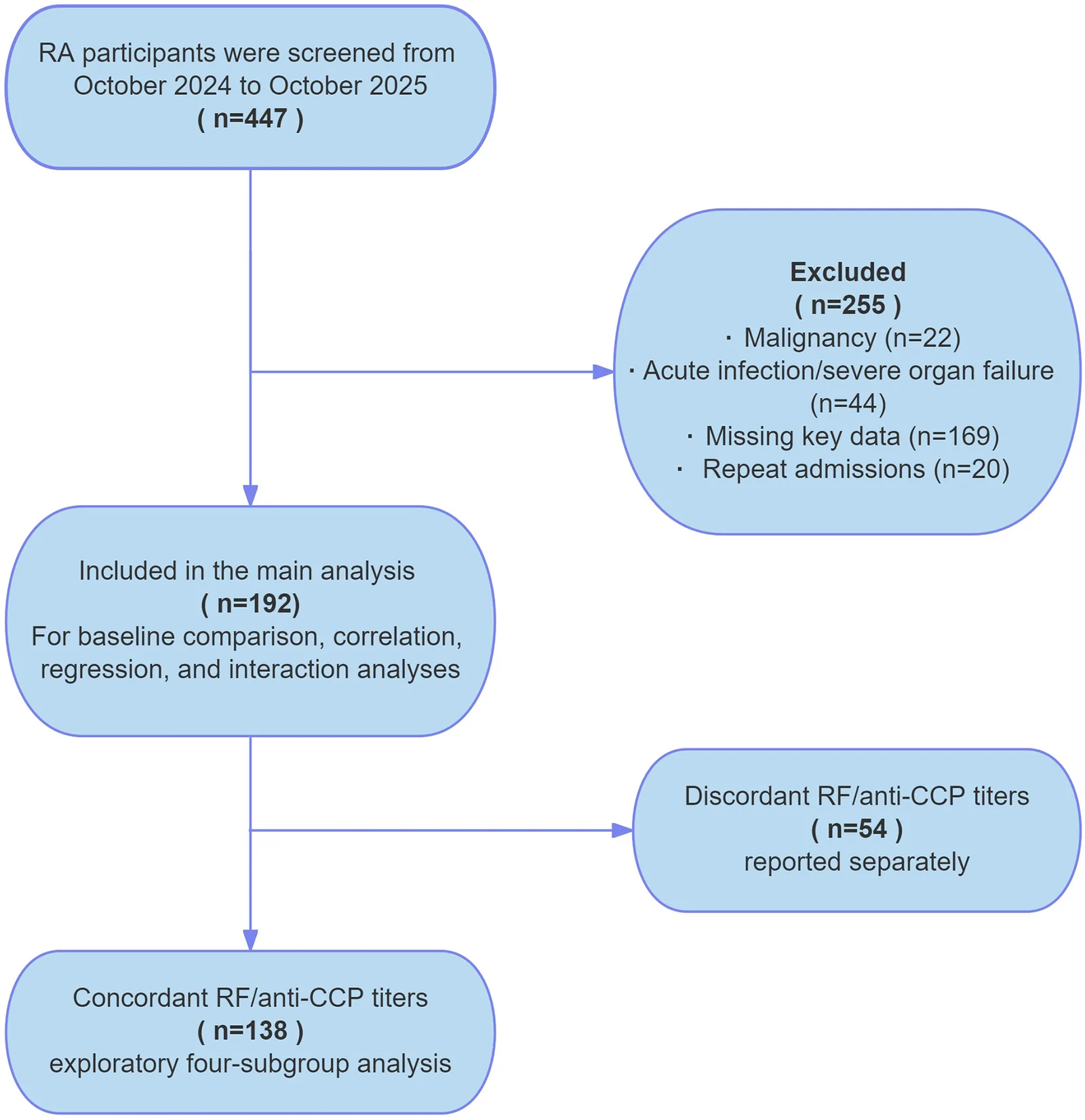
Flowchart of patient selection and grouping. A total of 447 hospitalized patients with confirmed rheumatoid arthritis were screened from October 2024 to October 2025; only the first admission record was retained for patients with multiple hospitalizations. Of these, 255 patients were excluded: 22 with malignancy, 44 with acute infection or severe organ failure, 169 with missing key clinical or laboratory data, and 20 with repeated admissions. The remaining 192 patients constituted the overall cohort for baseline comparison, correlation, regression and interaction analyses. Further exclusion of 54 patients with inconsistent RF/anti-CCP titers (their clinical characteristics are reported separately), and 138 patients with consistent titers were included in the exploratory four-stratification analysis.


$$\mathrm{DAS}28-\mathrm{ESR}=0.56\times \sqrt{\mathrm{TJC}28}+0.28\times \sqrt{\mathrm{SJC}28}+0.70\times \ln(\mathrm{ESR})+0.014\times \mathrm{VAS}.$$

where *TJC*28 and *SJC*28 represent the 28-joint tender and swollen joint counts, respectively, and Visual Analogue Scale(*VAS*) is the patient's global health assessment of disease activity on a 0–100 mm scale (0 = best, 100 = worst). Collected clinical variables included age, sex, body mass index (BMI), disease duration, glucocorticoid use, hypertension, and diabetes mellitus; diabetes was recorded as a general comorbidity, and no systematic screening was performed for other organ-specific autoimmune diseases (e.g., type 1 diabetes, Addison disease, vitiligo). Laboratory measurements and DAS28-ESR clinical assessment were both completed during hospitalization, with the time windows of clinical and laboratory data collection kept close.

This retrospective analysis relied solely on archived clinical records, with no additional interventions, examinations, or treatments performed on patients. Owing to the long time span of the records, written informed consent could not be obtained from every participant; all raw data were de-identified to protect patient privacy. The study protocol was approved by the Ethics Committee of the First Affiliated Hospital of Guizhou University of Traditional Chinese Medicine (approval no. KY2023-007A) with a waiver of informed consent, and was conducted in accordance with the principles of the Declaration of Helsinki.

### 2.2 Laboratory testing and subgroup stratification strategies

All patients underwent serum RF, anti-CCP, hs-CRP, and ESR measurements during hospitalization. TRAb and thyroid function tests (TSH, FT3, FT4) were ordered only for RA inpatients in whom thyroid disease was clinically suspected (e.g., goiter, symptoms suggestive of thyroid dysfunction, or a history of thyroid disease), rather than routinely for all RA patients; the potential impact of this selective testing indication on sample representativeness is addressed in the Discussion. TRAb was measured by electrochemiluminescence immunoassay (Roche Cobas e601 analyzer, second-generation TRAb assay), with 1.75 IU/L as the upper reference limit; TRAb ≥1.75 IU/L was defined as TRAb-positive (TRAb⁺) and <1.75 IU/L as TRAb-negative (TRAb⁻). RF and hs-CRP were measured by immunoturbidimetry, anti-CCP by chemiluminescence immunoassay, and ESR by the Westergren method. The reagent platforms, reference intervals, analytical measurement ranges, and handling of results outside the measurement range for each assay are detailed in Supplementary Material S2; the same platforms and methods were used throughout the study period.

Antibody titer stratification was performed as follows. RF and anti-CCP cutoffs were defined by their respective sample medians across the full cohort (RF 68.05 IU/mL; anti-CCP 83.50 U/mL): patients with both antibodies below their respective medians were defined as low titer, both at or above their respective medians as high titer, and one above with the other below as discordant. Because RF and anti-CCP differ in units and assay systems, separate medians were calculated for each antibody, and no single pooled median was used. The primary analysis always retained all 192 patients. Among these, the 138 patients with concordant antibody titers entered an exploratory four-group analysis stratified by TRAb status and antibody titer: TRAb⁻ + low titer, TRAb⁺ + low titer, TRAb⁻ + high titer, and TRAb⁺ + high titer. The 54 patients with discordant titers remained in the primary analysis cohort, were additionally described separately with respect to clinical characteristics, and were included in the interaction analyses based on all 192 patients.

### 2.3 Statistical analysis

Statistical analyses were performed using SPSS 26.0 and GraphPad Prism 9.0. The RCS analysis was performed using R software (version 4.4.1) with the rms package (version 6.8.0). Normality was assessed by the Shapiro–Wilk test. Normally distributed continuous variables were expressed as mean ± SD and compared with the independent-samples *t* test; non-normally distributed variables were expressed as median (IQR) and compared with the Mann–Whitney *U* test. Categorical variables were expressed as *n* (%) and compared using the *χ*^2^ test. Fisher exact test was used when the expected frequency was <5. Comparisons among multiple groups were performed using the Kruskal–Wallis *H* test, followed by Dunn's test with Bonferroni correction. Pairwise correlations were assessed using Spearman rank correlation (*ρ*). The dose–response relationship between TRAb and DAS28-ESR was evaluated using a restricted cubic spline (RCS) model (four knots at the 5th, 35th, 65th, and 95th percentiles), with the test for nonlinearity reported.

Multiple linear regression models were constructed with DAS28-ESR as the dependent variable; covariates included TRAb positivity, Ln(RF), Ln(anti-CCP), TSH, age, disease duration, BMI, and glucocorticoid use. RF and anti-CCP were natural-log transformed before entry into the models, and values below the lower limit of detection were handled as described in Supplementary Material S2. Multicollinearity was assessed using the variance inflation factor (VIF), and model adequacy was evaluated by residual analysis, tests for homoscedasticity, and examination of influential points. Because hs-CRP and ESR are both acute-phase inflammatory markers and ESR is a component of the DAS28-ESR score, hs-CRP was not included in the primary regression model (used only for descriptive comparisons). Interaction terms [TRAb  ×  Ln(RF) and TRAb  ×  Ln(anti-CCP)] were added separately to the primary model in all 192 patients. All analyses were based on all 192 patients; the primary analysis was repeated in the 167 euthyroid patients as a sensitivity analysis, and the four-group and interaction analyses were considered exploratory. All tests were two-sided, and *P* < 0.05 was considered statistically significant.

## 3 Result

### 3.1 Baseline clinical characteristics and thyroid function status

A total of 192 RA patients were included, comprising 51 TRAb⁺ and 141 TRAb⁻ patients. Age, sex distribution, and the proportions of hypertension and diabetes did not differ significantly between the two groups (all *P* > 0.05). Compared with the TRAb⁻ group, TRAb⁺ patients had longer disease duration (*Z* = 3.121, *P* = 0.002), lower BMI (*Z* = −3.886, *P* < 0.001), and a higher proportion of glucocorticoid use (*χ*^2^ = 9.706, *P* = 0.002) (Table 1).

**Table 1:** Comparison of baseline demographic, clinical characteristics, and thyroid function (*N* = 192).

Variable	TRAb^+^ group (*n* = 51)	TRAb^−^ group(*n* = 141)	*Z*/*χ*^2^	*P* value
Age, years	49.9 (46.0, 56.6)	46.8 (43.0, 62.0)	0.306	0.761
Male, *n* (%)	23 (45.1)	57 (40.4)	0.172	0.679
Disease duration, years	8.2 (6.0, 10.2)	6.8 (4.5, 9.6)	3.121	0.002
BMI, kg/m^2^	23.5 (21.7, 24.6)	24.3 (23.0, 26.0)	−3.886	<0.001
Glucocorticoid use, *n* (%)	35 (68.6)	59 (41.8)	9.706	0.002
Hypertension, *n* (%)	18 (35.3)	42 (29.8)	0.303	0.582
Diabetes mellitus, *n* (%)	9 (17.6)	20 (14.2)	0.132	0.716
TSH, mIU/L	1.4 (0.1, 1.9)	2.5 (2.0, 3.0)	−7.557	<0.001
FT3, pmol/L	5.0 (4.5, 5.8)	4.7 (4.3, 5.2)	2.805	0.005
FT4, pmol/L	17.8 (16.4, 20.2)	16.5 (15.1, 17.7)	4.006	<0.001
Euthyroidism, *n* (%)	36 (70.6)	131 (92.9)	—	—
Overt hyperthyroidism, *n* (%)	10 (19.6)	0 (0.0)	—	—
Subclinical hyperthyroidism, *n* (%)	5 (9.8)	1 (0.7)	—	—
Subclinical hypothyroidism, *n* (%)	0 (0.0)	8 (5.7)	—	—
Overt hypothyroidism, *n* (%)	0 (0.0)	1 (0.7)	—	—
Distribution of thyroid function status	—	—	*χ*^2^ = 42.961 (df = 4)	<0.001

Continuous variables are presented as median (interquartile range) and compared between groups using the Mann–Whitney U test (Z value reported); categorical variables are presented as *n* (%) and compared using the chi-square test (Fisher exact test when the expected frequency was <5). The distribution of thyroid function status was compared using the chi-square test with 4 degrees of freedom. “—” indicates not applicable.

With respect to thyroid function, TSH was lower (*Z* = −7.557, *P* < 0.001) and FT3 (*Z* = 2.805, *P* = 0.005) and FT4 (*Z* = 4.006, *P* < 0.001) were higher in the TRAb⁺ group than in the TRAb⁻ group (Table 1). According to thyroid functional status, 70.6% (36/51) of TRAb⁺ patients were euthyroid, 19.6% (10/51) had overt hyperthyroidism, and 9.8% (5/51) had subclinical hyperthyroidism; among TRAb⁻ patients, 92.9% (131/141) were euthyroid, and the remainder comprised one subclinical hyperthyroidism, eight subclinical hypothyroidism, and one overt hypothyroidism. The distribution of thyroid functional status differed significantly between the two groups (*χ*^2^ = 42.961, *df* = 4, *P* < 0.001). Thus, although the thyroid functional profile of TRAb⁺ patients differed from that of TRAb⁻ patients, the majority of TRAb⁺ patients remained euthyroid.

### 3.2 Immune-inflammatory indicators and DAS28-ESR by TRAb status

Among all 192 patients, the rank distributions of RF (*Z* = 3.624, *P* < 0.001) and anti-CCP (*Z* = 3.352, *P* < 0.001) differed significantly between the TRAb⁺ and TRAb⁻ groups; the inflammatory markers hs-CRP (*Z* = 9.624, *P* < 0.001) and ESR (*Z* = 9.161, *P* < 0.001) were significantly higher in the TRAb⁺ group. The median DAS28-ESR score was 6.1 (IQR 4.4, 6.7) in the TRAb⁺ group vs. 4.1 (IQR 2.9, 4.8) in the TRAb⁻ group (*Z* = 6.053, *P* < 0.001) (Table 2). Because the distributions of RF and anti-CCP were right-skewed, the medians were similar between the two groups whereas the high-titer end of the distribution differed (higher upper quartiles in the TRAb⁺ group); between-group differences were therefore interpreted on the basis of the rank test results.

**Table 2:** Comparison of immune-inflammatory parameters and disease activity.

Variable	TRAb^+^ group (*n* = 51)	TRAb^−^ group(*n* = 141)	*Z*	*P* value
RF, IU/mL	66.3 (53.4, 111.5)	69.8 (38.3, 86.9)	3.624	<0.001
anti-CCP, U/mL	81.4 (69.8, 138.2)	85.4 (50.0, 94.4)	3.352	<0.001
hs-CRP, mg/L	32.5 (18.9, 36.1)	12.4 (9.6, 16.2)	9.624	<0.001
ESR, mm/h	57.9 (52.7, 63.1)	28.7 (23.1, 40.9)	9.161	<0.001
DAS28-ESR	6.1 (4.4, 6.7)	4.1 (2.9, 4.8)	6.053	<0.001

Continuous variables are presented as median (interquartile range) and compared using the Mann–Whitney *U* test; *Z* is the test statistic.

### 3.3 Correlation analyses

The Spearman pairwise correlations among the study variables are shown in Figure 2. TRAb was positively correlated with RF (*ρ*=0.29), anti-CCP (*ρ*=0.29), and DAS28-ESR (*ρ*=0.36), and negatively correlated with TSH (*ρ*=−0.55). RF and anti-CCP were moderately positively correlated (*ρ*=0.47) and were each correlated with DAS28-ESR (*ρ*=0.53 and 0.52, respectively). FT3 and FT4 showed weak correlations with DAS28-ESR (*ρ*=0.17 and 0.20), whereas TSH was negatively correlated with DAS28-ESR (*ρ*=−0.33). Overall, TRAb and RA-specific autoantibodies were positively correlated with disease activity, whereas thyroid function-related indicators showed negative or weak correlations.

**Figure 2:**
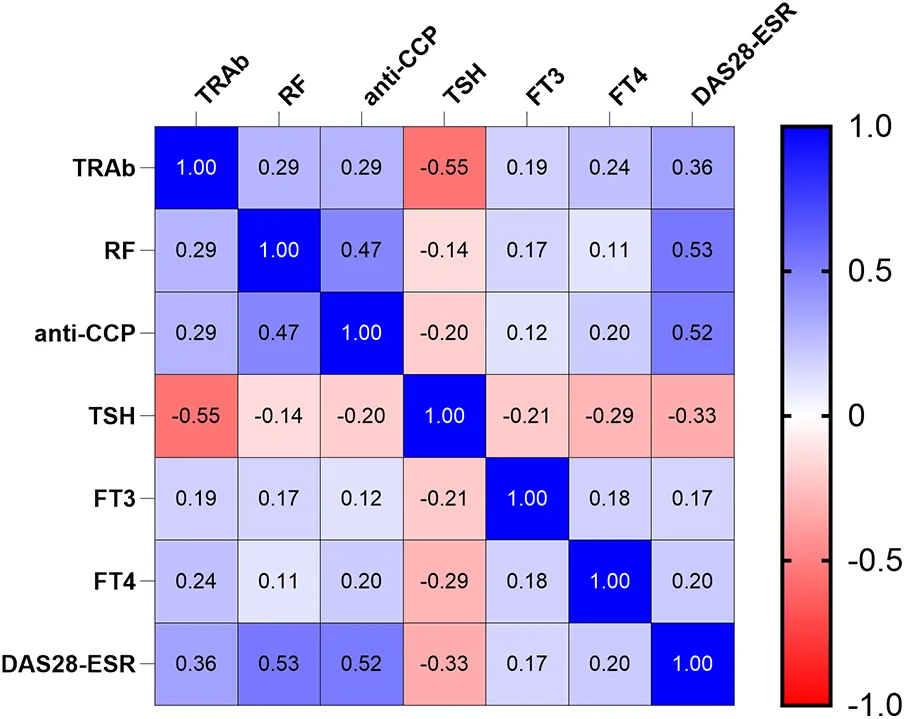
Heatmap of spearman pairwise correlations among study variables. Color intensity represents the Spearman correlation coefficient *ρ*, red indicates negative correlation and blue indicates positive correlation, with darker colors indicating stronger correlations.

### 3.4 Association between TRAb levels and DAS28-ESR

The RCS model revealed a significant overall association between TRAb and DAS28-ESR (*P* < 0.001) and a nonlinear dose-response relationship (test for nonlinearity *F* = 6.360, *P* = 0.002; Figure 3A). At the group level, DAS28-ESR scores were generally higher in TRAb⁺ than in TRAb⁻ patients (Figure 3B); individual TRAb⁺ patients with low DAS28-ESR scores (close to remission) were observed, reflecting inter-individual variability and indicating that not all TRAb⁺ patients were in a high disease activity state.

**Figure 3:**
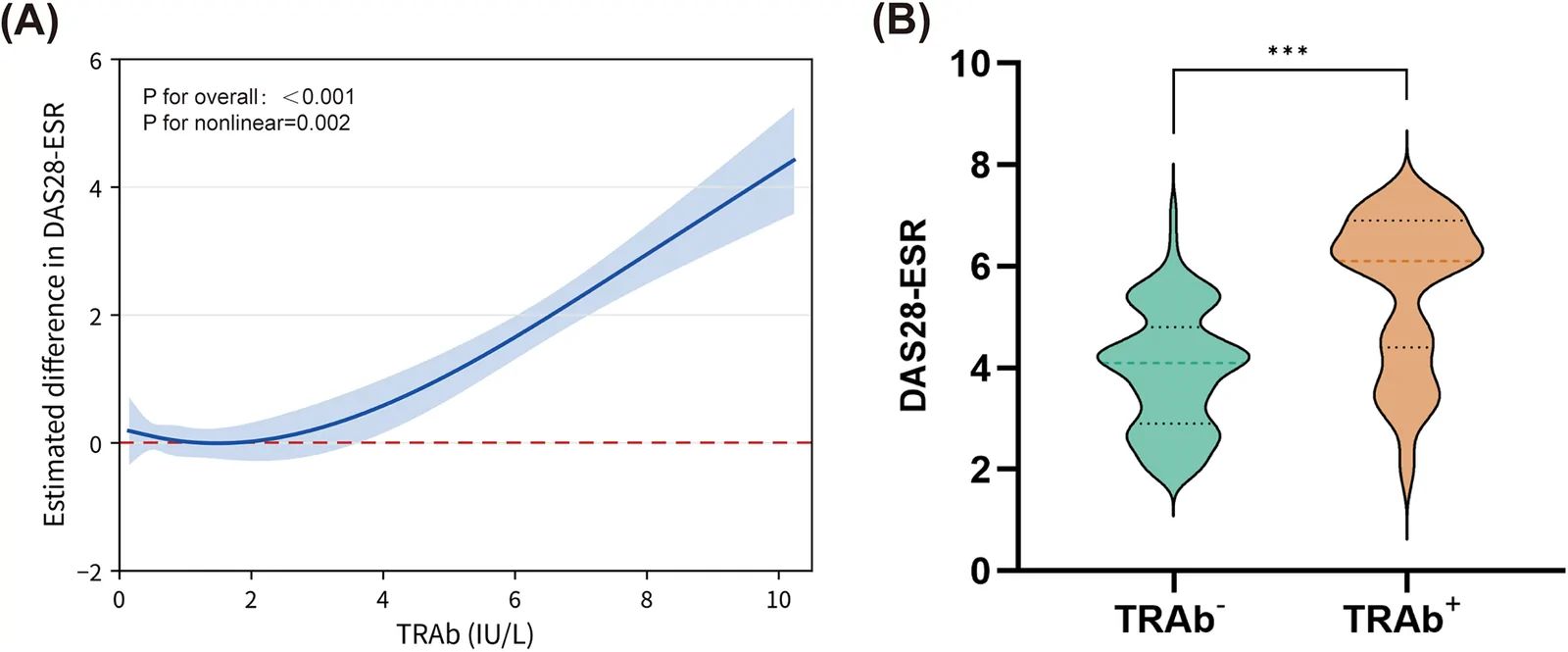
Association between TRAb and DAS28-ESR score. **(A)** Restricted cubic spline (RCS) model fitting the dose–response relationship between TRAb and DAS28-ESR (nonlinear test *P* = 0.002); **(B)** Violin plot of DAS28-ESR scores in the TRAb⁺ and TRAb⁻ groups; ***P < 0.001.

### 3.5 Multiple linear regression analysis of factors associated with DAS28-ESR

Results of the multivariable linear regression with DAS28-ESR as the dependent variable are shown in Table 3, which reports the unstandardized coefficient, standard error, standardized coefficient *β*, 95% CI, VIF, and test statistics for all covariates. All VIF values ranged from 1.03 to 2.24, indicating no significant multicollinearity; residual analysis and tests for homoscedasticity revealed no major violation of the model assumptions. After adjustment for TSH, age, disease duration, BMI, and glucocorticoid use, TRAb positivity was independently associated with DAS28-ESR (*B* = 1.457, 95% CI 1.008–1.906, *β*=0.643, *P* < 0.001). Ln(RF) (*B* = 0.412, *P* = 0.025) and Ln(anti-CCP) (*B* = 0.454, *P* = 0.025) were also independently associated with DAS28-ESR; TSH (*B* = −0.105, *P* = 0.023) and BMI (*B* = 0.338, *P* < 0.001) were significantly associated with DAS28-ESR. Age, disease duration, and glucocorticoid use were not statistically significant in this model. The overall model *R*^2^ was 0.577, adjusted *R*^2^ 0.558 [*F*(8,183) = 31.180, *P* < 0.001].

**Table 3:** Multivariable linear regression of factors associated with DAS28-ESR (*N* = 192).

Variable	*B*	*SE*	*β*	*t*	*P* value	95% CI	VIF
Constant	−7.405	1.287	—	−5.752	<0.001	(−9.928, −4.882)	—
TRAb	1.457	0.229	0.643	6.360	<0.001	(1.008, 1.906)	2.12
Ln(RF)	0.412	0.183	0.198	2.254	0.025	(0.054, 0.770)	1.60
Ln(anti-CCP)	0.454	0.201	0.202	2.261	0.025	(0.061, 0.848)	1.65
TSH, mIU/L	−0.105	0.045	−0.176	−2.302	0.023	(−0.194, −0.016)	1.21
Age, years	−0.004	0.008	−0.031	−0.442	0.659	(−0.020, 0.012)	1.03
Disease duration, years	−0.014	0.027	−0.036	−0.506	0.614	(−0.066, 0.039)	1.06
BMI, kg/m^2^	0.338	0.064	0.551	5.304	<0.001	(0.213, 0.463)	2.24
Glucocorticoid use	0.275	0.167	0.137	1.649	0.101	(−0.052, 0.601)	1.44

Primary analysis model with DAS28-ESR as the dependent variable; RF and anti-CCP were natural-log transformed before entry. Unstandardized coefficient (*B*), standard error (*SE*), standardized coefficient (*β*), *t* value, *P* value, 95% confidence interval (CI), and variance inflation factor (VIF) are presented for all covariates. Model *R*^2^ = 0.577, adjusted *R*^2^ = 0.558, *F*(8,183) = 31.180, *P* < 0.001; all VIFs <2.3, indicating no significant collinearity.

### 3.6 Sensitivity analysis in the euthyroid subgroup

To evaluate whether the observed associations were driven by abnormal thyroid function, the primary analysis was repeated in the 167 euthyroid patients (Supplementary Table S1, S2). In the TRAb⁺ group (*n* = 36), hs-CRP (26.1 vs. 12.7 mg/L, *P* < 0.001), ESR (55.0 vs. 28.9 mm/h, *P* < 0.001), and DAS28-ESR (4.9 vs. 4.1, *P* < 0.001) remained higher than in the TRAb⁻ group (*n* = 131); TRAb positivity remained associated with DAS28-ESR in the multivariable regression (*B* = 1.312, 95% CI 0.756–1.869, *P* < 0.001). These results indicate that the association between TRAb and disease activity persisted after excluding clinical and subclinical thyroid dysfunction.

### 3.7 Exploratory four-group analysis by TRAb status and antibody titer, and interaction analyses

After stratification by TRAb status and antibody titer, the median (IQR) DAS28-ESR scores were 2.8 (2.5, 3.6) in the TRAb⁻ low-titer group (A, *n* = 49), 3.7 (3.3, 4.6) in the TRAb⁺ low-titer group (B, *n* = 20), 4.3 (3.5, 5.4) in the TRAb⁻ high-titer group (C, *n* = 54), and 6.9 (6.7, 7.1) in the TRAb⁺ high-titer group (D, *n* = 15) (Figure 4). The Kruskal–Wallis *H* test showed a statistically significant difference across the four groups (*H* = 64.277, *df* = 3, *P* < 0.001). Pairwise comparisons by Dunn's test with Bonferroni correction showed that, within the low-titer stratum, DAS28-ESR was higher in TRAb⁺ than in TRAb⁻ patients (adjusted *P* = 0.021); within the high-titer stratum, TRAb⁺ patients had higher scores than TRAb⁻ patients (adjusted *P* < 0.001); irrespective of TRAb status, high-titer groups had higher scores than low-titer groups of the same TRAb status (both adjusted *P* < 0.001); and the difference between the TRAb⁻ high-titer group and the TRAb⁺ low-titer group was not statistically significant (adjusted *P* = 1.000). These findings suggest a titer-dependent association between TRAb and disease activity, with the highest disease activity observed in patients with concurrent TRAb positivity and high-titer RA autoantibodies.

**Figure 4:**
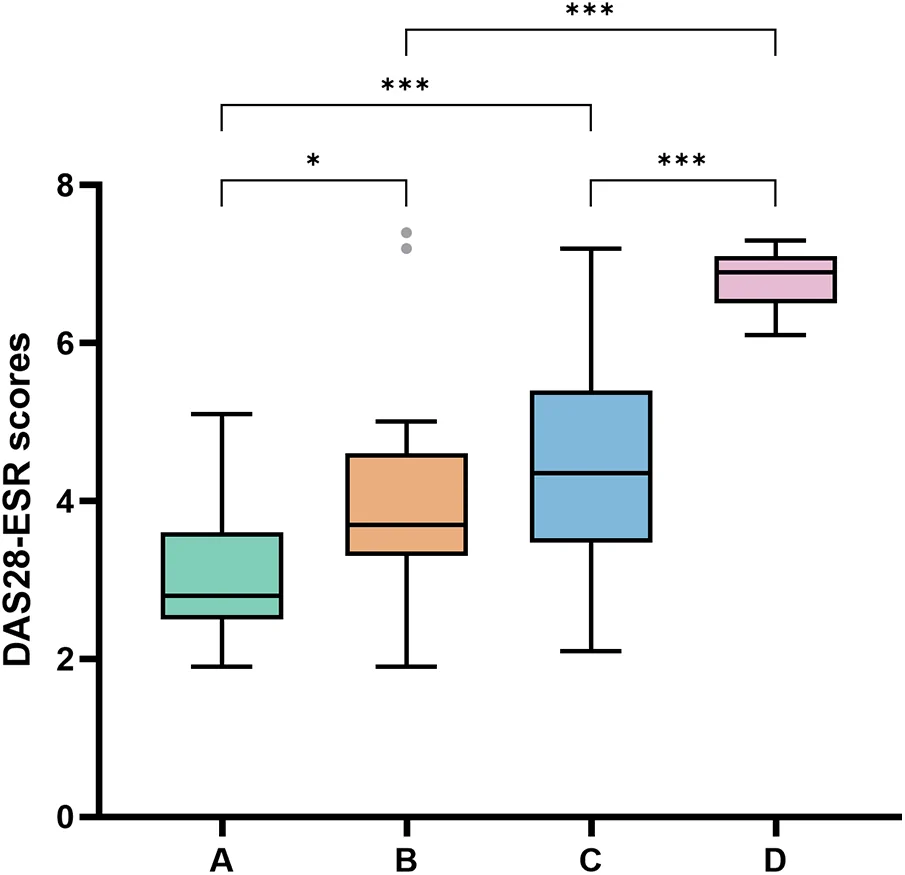
Comparison of *DAS28-ESR* scores across subgroups stratified by TRAb status and autoantibody titers. Boxes represent the interquartile range (Q1–Q3), the horizontal line inside each box is the median, and whiskers are plotted by Tukey's method. Sample sizes of four subgroups: TRAb⁻ low titer (A, *n* = 49), TRAb⁺ low titer (B, *n* = 20), TRAb⁻ high titer (C, *n* = 54), TRAb⁺ high titer (D, *n* = 15). The overall between-group difference was assessed by Kruskal–Wallis *H* test (*H* = 64.277, *df* = 3, *P* < 0.001). Pairwise comparisons were performed using Dunn's test with Bonferroni correction. Significance markers indicate *adjusted P* values after Bonferroni correction. ns, *adjusted P* > 0.05; *, *adjusted P* < 0.05; ***, *adjusted P* < 0.001.

The 54 patients with discordant titers were reported separately (Supplementary Table S3): 38 were TRAb⁻ and 16 were TRAb⁺, with median DAS28-ESR scores of 4.3 (IQR 4.2, 5.0) and 6.2 (IQR 6.1, 6.3), respectively; their disease activity lay between the corresponding phenotypes of the concordant population. Of note, the four-group analysis is based on the Kruskal–Wallis test and pairwise comparisons, which describe differences in disease activity between groups and do not directly test interaction. To formally evaluate the statistical interaction between TRAb and RA autoantibodies, product terms were added to the primary analysis model in all 192 patients: the TRAb  ×  Ln(anti-CCP) interaction was significant (*B* = 1.360, 95% CI 0.538–2.182, *P* = 0.0014), and the TRAb  ×  Ln(RF) interaction was borderline significant (*B* = 0.746, 95% CI 0.018–1.475, *P* = 0.046) (Supplementary Table S4). These findings suggest that the TRAb–DAS28-ESR association strengthened with increasing rheumatoid autoantibody titers. Statistical interaction indicates that the strength of an association varies across levels of another variable and, in this cross-sectional design, does not permit inference of biological synergy or causal mechanisms; the four-group and interaction analyses should thus be considered exploratory.

## 4 Discussion

This single-center retrospective cross-sectional study of 192 hospitalized patients with RA observed that TRAb positivity was associated with higher disease activity and greater systemic inflammation, an association that persisted after adjustment for RF and anti-CCP titers and thyroid function indicators and was reproducible in euthyroid patients. When patients were stratified by TRAb status and autoantibody titers, DAS28-ESR increased in a stepwise manner across the four subgroups, with the highest disease activity observed in TRAb⁺ patients with high-titer rheumatoid autoantibodies. Interaction testing revealed a statistically significant interaction between TRAb and Ln(anti-CCP) (*P* = 0.0014) and a borderline-significant interaction with Ln(RF) (*P* = 0.046), suggesting that the strength of the TRAb–DAS28-ESR association depends on rheumatoid autoantibody titers. It should be emphasized that this was a retrospective cross-sectional study that can only reveal associations; biological synergy or causal inflammatory mechanisms cannot be inferred from the present data.

Breakdown of immune tolerance typically manifests as autoimmune comorbidity and the coexistence of multiple autoantibodies. Genetic susceptibility loci, innate immune dysfunction and adaptive immune disorder jointly stimulate the production of polyclonal autoantibodies (20, 21). RA and AITDs share multiple core susceptibility genes, including the HLA-DRB1 shared epitope (22, 23), PTPN22 (24, 25), CTLA-4 (26, 27), and STAT4 (28, 29). These genes regulate T-cell activation, B-cell receptor signaling, and immune checkpoint function, thereby increasing the risk of developing systemic and organ-specific autoimmune responses simultaneously. To a certain extent, the clustering of RA and thyroid autoimmunity can be understood within the broader framework of polyglandular/polyautoimmune phenotypes. Autoimmune polyendocrine syndrome type 3 (APS-3), whose defining feature is the coexistence of AITD with other autoimmune diseases, is a notable example of this framework; it is not rare in the general population and carries a complex genetic background (30). Although RA is not a classic component of APS-3, the clustering of RA and AITD in the same individual follows a similar polyautoimmune pattern. Environmental factors, such as smoking, chronic infection, and sex hormone fluctuations, further disrupt immune tolerance through molecular mimicry and epigenetic modifications, activating polyclonal B cells and inducing autoantibodies against distinct tissues, including the joints and the thyroid (17, 31, 32). In this context, the high TRAb positivity rate in RA patients may reflect a shared autoimmune susceptibility state rather than a simple complication of thyroid dysfunction. Previous studies generally regarded TRAb positivity in RA merely as a secondary serological feature of GD, focusing on the indirect influence of thyroid dysfunction on RA progression while overlooking the potential role of TRAb within this broader autoimmune network.

Direct evidence supporting extrathyroidal actions of TRAb remains limited. Studies on the local thyroid hormone network (deiodinases and thyroid hormone receptors TR*α*/TR*β*) have described hormone-sensitivity regulation in various tissues (33), and TSH has been reported to modulate immune-related communication through extracellular vesicles derived from hepatocytes (34); however, neither line of evidence involves TSHR expression in synovial or immune cells and therefore cannot serve as evidence for functional intra-articular TSHR signaling. Similarly, studies of other thyroid autoantibodies, suggest that thyroid-directed autoimmunity can influence systemic inflammatory states, yet these findings involve antibodies other than TRAb or receptor systems other than TSHR (35, 36). Thus, although these observations collectively support a link between thyroid autoimmunity and RA inflammation, direct human evidence that TRAb itself mediates synovial inflammation is still lacking, and any TSHR-related mechanism in RA remains hypothetical. Moreover, the present study measured only total TRAb and could not distinguish stimulatory, blocking, or neutral subtypes, which further limits mechanistic interpretation. In our cohort, TRAb showed low-to-moderate positive correlations with RF and anti-CCP, and TRAb positivity remained independently associated with disease activity after adjustment for rheumatoid autoantibodies, suggesting that the association is not fully mediated by rheumatoid autoantibody levels; interaction testing further indicated that the strength of the TRAb–disease activity association increased with antibody titers. These findings position TRAb positivity as a marker within a broader autoimmune context rather than an isolated thyroid abnormality, although the underlying mechanisms warrant functional studies.

At the clinical phenotype level, previous cohort studies have yielded inconsistent conclusions regarding outcomes in TRAb⁺ RA patients (37). Rahmouni et al. (38)reported a TRAb positivity rate of 3.4% in an RA cohort and found no significant association between thyroid autoantibody status and DAS28 or CRP; the TRAb positivity rate in our study (26.6%) was substantially higher. This difference may reflect differences in study population composition, TRAb testing indications, and assay systems. In particular, TRAb measurement in our research was mainly ordered for hospitalized patients with clinically suspected thyroid disease, which may have enriched the sample for individuals with autoimmune thyroid predisposition and thus inflated the positivity rate. Discrepancies among previous findings may also be influenced by sample size, TRAb cut-off values, whether isolated TRAb positivity was distinguished from overt hyperthyroidism, and whether rheumatoid autoantibody titers were adjusted. Most prior studies adopted simple binary grouping by TRAb status without titer stratification, which may obscure associations that emerge only when antibody levels are taken into account.

From a clinical perspective, RF and anti-CCP are routinely used for the diagnosis and prognostic evaluation of RA, whereas TRAb has not been incorporated into routine RA screening. Our results suggest that TRAb⁺ RA patients have higher systemic inflammation even when thyroid function is normal, and that TRAb positivity combined with high-titer RF/anti-CCP represents a phenotype with a higher inflammatory burden that warrants clinical attention (39). It should be emphasized that this was an exploratory cross-sectional analysis; the incremental clinical value of incorporating TRAb into RA stratification requires validation in prospective cohorts through comparison with baseline models that do not include TRAb.

### 4.1 Limitations

This study has several limitations. First, the single-center retrospective cross-sectional design may introduce selection bias, and caution is warranted when generalizing the findings. Second, TRAb and thyroid function testing were performed only in hospitalized patients with clinically suspected thyroid disease, which may have enriched the sample with individuals prone to thyroid autoimmunity and partly explains the higher TRAb positivity rate in this study (26.6%) than the previously reported 3.4%. Third, cross-sectional studies can only evaluate associations; the four-subgroup analysis and interaction tests were exploratory, and a statistically significant interaction does not imply biological synergy. Moreover, the TRAb-positive high-titer subgroup was small (*n* = 15), limiting the precision of the interaction estimates. Fourth, only total TRAb was measured; stimulating, blocking, and neutral subtypes were not distinguished, and anti-TPO and anti-thyroglobulin antibodies were not assessed, making it difficult to fully separate TRAb-specific effects from the broader background of thyroid autoimmunity. In addition, immunoassay interference cannot be entirely excluded in RF-positive samples. Fifth, owing to the retrospective nature of the data, glucocorticoid use was classified only as present or absent; DMARD history and potential confounders such as smoking were not adjusted for, and the individual joint-count components of DAS28 and CDAI were not reported. Sixth, the interval between laboratory measurements and clinical assessments could not be strictly defined. Seventh, the RF and anti-CCP titer cut-offs were based on the sample medians of this cohort, representing a data-driven stratification whose generalizability remains to be validated; the incremental clinical value of combined TRAb testing also warrants prospective evaluation. Despite these limitations, this study provides supplementary evidence for the autoimmune comorbidity of RA and AITD based on real-world data.

## 5 Conclusion

TRAb positivity was associated with higher disease activity and systemic inflammation in patients with RA, and this association persisted in euthyroid patients; moreover, the strength of the association increased with higher titers of rheumatoid autoantibodies. The value of combined testing of TRAb, RF, and anti-CCP for immune stratification in RA warrants prospective validation.

## Data Availability

The datasets presented in this article are not readily available because raw individual-level patient data cannot be publicly released owing to ethical and patient-privacy restrictions. Qualified researchers may request de-identified minimal datasets from the corresponding author following institutional ethical approval. Aggregated data are fully available within this manuscript. Requests to access the datasets should be directed to Hongwei He, 1573773291@qq.com.
