# Supplementary material for: Associations between thyrotropin receptor antibody, rheumatoid autoantibodies, and disease activity in patients with rheumatoid arthritis: a retrospective cross-sectional study
Source: Front Med (Lausanne). 2026 Oct 21;13:1970456. doi: 10.3389/fmed.2026.1970456 (PMC13730959; doi:10.3389/fmed.2026.1970456)
Supplement: Supplementary file 1 [file Supplementaryfile1.pdf]

STROBE Statement—Checklist of items that should be included in reports of ***cross-sectional studies***

	Item No	Recommendation
**Title and abstract**	1	(*a*) Both the title and abstract clearly states this article as“retrospective cross-sectional study”.
		(*b*) A structured abstract systematically covers research objectives, methodology, baseline participant characteristics, correlation and stratified synergistic analyses, and conclusions, fully summarizing the entire research process and primary findings
**Introduction**		
Background/rationale	2	We illustrate the clinical background of comorbidity between rheumatoid arthritis (RA) and autoimmune thyroid diseases. Thyroid-stimulating hormone receptors are expressed on synovial cells. Existing literature only separately investigates TRAb, lacking evidence regarding the synergistic effect of TRAb combined with RF and anti-CCP on disease activity.
Objectives	3	Study objectives: 1) Analyze correlations between TRAb and RF/anti-CCP in RA patients; 2) Clarify whether TRAb acts as an independent influencing factor for DAS28-ESR; 3) Verify that TRAb combined with high titers of rheumatoid-associated autoantibodies exerts a synergistic pro-inflammatory effect (prespecified hypothesis).
**Methods**		
Study design	4	The study is clearly defined as a single-center retrospective cross-sectional study at the outset. No prospective follow-up was performed; only archived inpatient medical records and laboratory results were retrieved without any clinical interventions.
Setting	5	Study site: The First Affiliated Hospital of Guizhou University of Traditional Chinese Medicine. Data extraction period: October 2024 to October 2025. No follow-up was conducted; laboratory tests were collected at different time points during each patient’s hospitalization.
Participants	6	(*a*) Inclusion criteria: Patients meeting the 2010 ACR/EULAR RA classification criteria with complete laboratory data of TRAb, RF, anti- CCP, hs- CRP and ESR to calculate DAS28- ESR. Participants were consecutively screened from hospitalized RA medical records. General exclusion criteria: coexistence of other connective tissue diseases or missing core clinical data. An additional 54 patients with discordant RF and anti- CCP results were excluded for stratified subgroup analysis.
Variables	7	Exposure variable: TRAb status (positive when ≥1.75 IU/L); predictive variables: RF and anti- CCP titers; outcome variable: DAS28- ESR disease activity score; confounding variables: age, sex, disease duration, BMI, glucocorticoid usage, hypertension, diabetes mellitus. RA diagnosis followed the 2010 ACR/EULAR classification criteria.
Data sources/ measurement	8*	All laboratory indicators were uniformly detected by automatic instruments in the hospital clinical laboratory with fixed reference ranges. Detection protocols for TRAb, RF and anti- CCP were identical across all subgroups. DAS28- ESR was calculated following a standardized unified procedure.
Bias	9	1) Consecutive enrollment reduced selection bias; 2) Multivariate linear regression adjusted for demographic and clinical confounders; 3) Stratified subgroup analysis controlled for confounding from autoantibody titers; 4) All data were de- identified to eliminate information bias; 5) Unified testing standards minimized measurement bias; residual confounding bias was discussed in the Limitations section.

Study size	10	All consecutive inpatients meeting diagnostic criteria within a one-year single-center period were enrolled without pre-planned sample size calculation for power analysis. The total initial cohort included 192 patients, and 138 patients remained for stratified subgroup analysis.
Quantitative variables	11	Skewed continuous variables were presented as median (interquartile range). RF, anti-CCP and hs-CRP were natural log-transformed before regression modeling. TRAb was dichotomized based on the laboratory cut-off value.Rheumatoid-associated autoantibodies were split into low/high titer subgroups using the cohort median for four-group stratified interaction analysis.
Statistical methods	12	(*a*) Statistical software: SPSS 26.0 and GraphPad Prism 9.0. Spearman rank correlation, Kruskal-Wallis H test, and multivariate linear regression adjusted for confounders were applied. VIF was calculated to test multicollinearity. All statistical tests were two-sided with $\alpha = 0.05$.
		(*b*) Four subgroups were established by cross-classifying TRAb status (negative/positive) and rheumatoid-associated autoantibody titer (low/high). Pairwise comparisons of DAS28-ESR across subgroups were performed to identify synergistic effects between the two antibody types.
		(*c*) Only patients with complete sets of laboratory tests were enrolled; no missing data existed in the final analytical dataset.
		(*d*) Consecutive retrospective sampling was adopted without complex stratified sampling; no weighting correction was required for analysis.
		(*e*) No sensitivity analyses were conducted, which is clearly stated in the manuscript.
**Results**		
Participants	13*	(a) A total of 192 consecutive hospitalized RA patients were initially screened. All met general inclusion criteria without preliminary exclusion. For stratified analysis, 54 patients with discordant RF/anti-CCP results were excluded, leaving 138 patients for four-subgroup analysis. The initial cohort contained 51 TRAb-positive and 141 TRAb-negative subjects. Results Section 1; Supplementary Material 2 Narrative of Participant
		(b) No patients were excluded for failing RA diagnostic criteria, concomitant other connective tissue diseases, or missing core data. The 54 excluded cases had inconsistent RF and anti-CCP serology, removed to avoid ambiguous autoantibody subgroup definition.
		(c) No flow diagram was included. A full textual description of participant screening was provided in the main Methods section.
Descriptive data	14*	(a) Table 1 reports baseline demographic and clinical characteristics (age, sex, disease duration, BMI, glucocorticoid use, comorbidities) stratified by TRAb status.
		(b) No missing demographic, laboratory or scoring variables were observed among all enrolled patients.
Outcome data	15*	Table 2 compares median values of RF, anti-CCP, hs-CRP, ESR and DAS28-ESR between TRAb subgroups. Figure 3 illustrates DAS28-ESR distribution across four subgroups with all intergroup P-values presented.
Main results	16	(*a*) Unadjusted intergroup comparisons were presented in descriptive results. Multivariate linear regression adjusted for age, disease duration, BMI, glucocorticoid therapy, hypertension and diabetes provided adjusted regression coefficients (B), standardized β and P-values (Table 3).
		(*b*) TRAb cut- off value: 1.75 IU/L (hospital laboratory standard). The cohort

		combined median of RF and anti- CCP was used as the boundary separating low and high autoantibody titers.
		(*c*) Not applicable; the study focused on linear associations without relative risk metrics.
Other analyses	17	Spearman rank correlation analyses evaluated associations between TRAb, inflammatory markers, autoantibodies and DAS28- ESR. Four- group stratified interaction analysis was performed to verify synergistic pro- inflammatory effects.
**Discussion**		
Key results	18	The opening paragraph of Discussion summarizes three core findings corresponding to the three research aims, interpreting the independent effect of TRAb and synergistic effects of dual autoantibodies.
Limitations	19	Limitations include single-center selection bias, retrospective laboratory testing at heterogeneous time points, undifferentiated TRAb functional subtypes, lack of follow-up data to evaluate joint damage and treatment response, and inability to establish causal inference due to cross-sectional design. Potential overestimation of inflammatory associations from residual bias is discussed.
Interpretation	20	Findings were cautiously interpreted by integrating basic mechanistic evidence of synovial TSHR expression, published population cohort data, and stratified interaction results from this study. Independent and synergistic inflammatory effects were distinguished without overstating causal conclusions.
Generalisability	21	The cohort consisted only of hospitalized RA patients from a single center; conclusions may not be generalizable to mild outpatient RA or populations from other regions. Multi-center cohorts are required for external validation.
**Other information**		
Funding	22	This study was supported by the National Natural Science Foundation of China (Grant No. 82360988)， the Joint Medical Research Project of Qianxinan Prefecture (Grant No. 2024-7), and the Postgraduate Education Innovation Program of Guizhou University of Traditional Chinese Medicine (Grant No. YCXKYB2025010).

*Give information separately for exposed and unexposed groups.

Note: An Explanation and Elaboration article discusses each checklist item and gives methodological background and published examples of transparent reporting. The STROBE checklist is best used in conjunction with this article (freely available on the Web sites of PLoS Medicine at http://www.plosmedicine.org/, Annals of Internal Medicine at http://www.annals.org/, and Epidemiology at http://www.epidem.com/). Information on the STROBE Initiative is available at www.strobe-statement.org.
