# Supplementary material for: Associations between thyrotropin receptor antibody, rheumatoid autoantibodies, and disease activity in patients with rheumatoid arthritis: a retrospective cross-sectional study
Source: Front Med (Lausanne). 2026 Oct 21;13:1970456. doi: 10.3389/fmed.2026.1970456 (PMC13730959; doi:10.3389/fmed.2026.1970456)
Supplement: Supplementary file 2 [file Table1.docx]

**Supplementary Material S2: Details of laboratory assays and quality‑control procedures**

# All laboratory tests were performed in the clinical laboratory of The First Affiliated Hospital of Guizhou University of Traditional Chinese Medicine. Analytical platforms, reagent kits and test methodologies remained identical throughout the whole study period. Assay calibration and daily internal quality control were implemented following manufacturer‑recommended standard operating procedures. No measurements exceeding the upper limit of quantification were observed in this cohort. Values below the lower limit of quantification (LLOQ) were substituted with LLOQ before natural‑logarithm transformation for statistical modelling, consistent with the pre‑specified statistical plan in the main manuscript.

| **Analyte** | **Assayprinciple** | **Instrument** | **Manufacturer‑providedreferenceinterval** | **Analyticalmeasuringrange** | **LLOQvalue** | **Internalquality‑control** |
| --- | --- | --- | --- | --- | --- | --- |
| TRAb | Second‑generation electrochemiluminescence immunoassay | Roche Cobas e601, Roche Diagnostics, Germany | ＜1.75 IU/L (negative); ≥1.75 IU/L (positive) | 0.10–40.00 IU/L | 0.10 IU/L | Daily run |
| RF | Immunoturbidimetry | Automated clinical chemistry analyser | 0–20 IU/mL | 10–800 IU/mL | 10 IU/mL | Daily run |
| anti‑CCP | Chemiluminescence immunoassay | Matched chemiluminescence platform | ＜20 U/mL (negative); ≥20 U/mL (positive) | 5.00–500.00 U/mL | 5.00 U/mL | Daily run |
| hs‑CRP | Immunoturbidimetry | Automated clinical chemistry analyser | 0–3.00 mg/L | 0.10–200.00 mg/L | 0.10 mg/L | Per‑batch run |
| ESR | Westergren method | Standard ESR analytical system | Female: 0‑20 mm/h; Male: 0‑15 mm/h | Not applicable | Not applicable | Per‑batch run |
| TSH | Electrochemiluminescence immunoassay | Roche Cobas e601, Roche Diagnostics, Germany | 0.27–4.20 mIU/L | Follow kit specification | Follow kit specification | Daily run |
| FT3 | Electrochemiluminescence immunoassay | Roche Cobas e601, Roche Diagnostics, Germany | 3.10–6.80 pmol/L | Follow kit specification | Follow kit specification | Daily run |
| FT4 | Electrochemiluminescence immunoassay | Roche Cobas e601, Roche Diagnostics, Germany | 12.00–22.00 pmol/L | Follow kit specification | Follow kit specification | Daily run |

LLOQ = lower limit of quantification. For RF and anti‑CCP, LLOQ values were assigned to specimens with readings below quantification limit prior to natural‑logarithm transformation for regression analyses. No samples above upper analytical measuring range were encountered in the present study cohort. Pre‑analytical sample handling complied with institutional laboratory standard operating procedures.

**Supplementary Tables**

**Supplementary Table S1. Comparison of baseline and immune-inflammatory parameters between the two groups in the euthyroid subgroup (sensitivity analysis) (n=167)**

| **Variable** | **TRAb^+^ group (*n*=36)** | **TRAb^-^ group (*n*=131)** | ***Z*/*χ*²** | ***P* value** |
| --- | --- | --- | --- | --- |
| Age, years | 52.1 (46.2, 57.0) | 47.2 (43.1, 62.0) | 0.054 | 0.958 |
| Disease duration, years | 8.6 (6.5, 10.3) | 6.7 (4.5, 9.1) | 3.257 | 0.001 |
| BMI, kg/m² | 22.2 (21.4, 23.6) | 24.3 (23.0, 25.9) | -5.781 | <0.001 |
| Male, n (%) | 11 (30.6) | 51 (38.9) | 0.528 | 0.468 |
| Glucocorticoid use, n (%) | 21 (58.3) | 56 (42.7) | 2.169 | 0.141 |
| Hypertension, n (%) | 13 (36.1) | 37 (28.2) | 0.577 | 0.448 |
| Diabetes mellitus, n (%) | 7 (19.4) | 18 (13.7) | 0.221 | 0.638 |
| TSH, mIU/L | 1.7 (1.3, 2.0) | 2.4 (2.0, 2.9) | -5.330 | <0.001 |
| FT3, pmol/L | 4.8 (4.3, 5.3) | 4.7 (4.3, 5.2) | 0.508 | 0.613 |
| FT4, pmol/L | 17.1 (15.1, 17.9) | 16.5 (15.1, 17.6) | 1.347 | 0.179 |
| RF, IU/mL | 59.8 (51.1, 94.3) | 69.8 (38.8, 86.9) | 1.277 | 0.203 |
| Anti-CCP, U/mL | 75.6 (67.2, 93.0) | 85.0 (49.6, 94.5) | 1.463 | 0.144 |
| hs-CRP, mg/L | 26.1 (17.9, 32.8) | 12.7 (9.6, 16.4) | 7.994 | <0.001 |
| ESR, mm/h | 55.0 (42.1, 58.8) | 28.9 (23.6, 41.3) | 7.457 | <0.001 |
| DAS28-ESR | 4.9 (3.7, 6.2) | 4.1 (3.0, 4.8) | 3.614 | <0.001 |

Only euthyroid patients (n=167) were included; statistical methods were the same as those for Table 1.

**Supplementary Table S2. Multivariable linear regression of factors associated with DAS28-ESR in the euthyroid subgroup (sensitivity analysis) (n=167)**

| Variable | *B* | *SE* | *β* | *t* | *P* value | 95% CI | VIF |
| --- | --- | --- | --- | --- | --- | --- | --- |
| Constant | -6.991 | 1.515 | — | -4.614 | <0.001 | (-9.984, -3.999) | — |
| TRAb-positive | 1.312 | 0.284 | 0.453 | 4.622 | <0.001 | (0.756, 1.869) | 2.39 |
| Ln(RF) | 0.424 | 0.195 | 0.214 | 2.175 | 0.031 | (0.042, 0.806) | 1.48 |
| Ln(anti-CCP) | 0.404 | 0.218 | 0.192 | 1.853 | 0.066 | (-0.023, 0.831) | 1.59 |
| TSH, mIU/L | -0.158 | 0.116 | -0.091 | -1.363 | 0.175 | (-0.386, 0.069) | 1.24 |
| Age, years | -0.002 | 0.009 | -0.012 | -0.186 | 0.853 | (-0.019, 0.015) | 1.04 |
| Disease duration, years | -0.014 | 0.029 | -0.032 | -0.470 | 0.639 | (-0.070, 0.043) | 1.07 |
| BMI, kg/m² | 0.330 | 0.077 | 0.438 | 4.300 | <0.001 | (0.179, 0.480) | 2.74 |
| Glucocorticoid use | 0.212 | 0.178 | 0.084 | 1.188 | 0.237 | (-0.138, 0.562) | 1.39 |

Only euthyroid patients (*n*=167) were included; model specifications were the same as those for Table 3, with *R*²=0.462, adjusted *R*²=0.435, *F*(8,158)=16.952, *P*<0.001. The association between TRAb positivity and DAS28-ESR remained robust in this subgroup.

**Supplementary Table S3. Clinical characteristics of the 54 patients with discordant RF/anti-CCP titers by TRAb status (exploratory analysis)**

| **Variable** | **TRAb^+^ group (*n*=16)** | **TRAb^-^ group (*n*=38)** | ***Z*/*χ*²** | ***P* value** |
| --- | --- | --- | --- | --- |
| Age, years | 50.8 (42.6, 54.9) | 52.3 (43.7, 61.9) | -1.345 | 0.182 |
| Disease duration, years | 7.0 (5.9, 9.1) | 7.0 (4.7, 9.2) | 1.241 | 0.218 |
| BMI, kg/m² | 23.6 (23.3, 24.0) | 24.6 (24.3, 24.7) | -4.783 | <0.001 |
| Male, n (%) | 8 (50.0) | 22 (57.9) | 0.054 | 0.816 |
| Glucocorticoid use, n (%) | 14 (87.5) | 25 (65.8) | 10.747 | 0.001 |
| Hypertension, n (%) | 10 (62.5) | 24 (63.2) | 0.000 | 1.000 |
| Diabetes mellitus, n (%) | 1 (6.3) | 2 (5.3) | 0.000 | 1.000 |
| TSH, mIU/L | 1.5 (1.2, 1.9) | 2.5 (2.1, 3.0) | -4.158 | <0.001 |
| RF, IU/mL | 115.1 (52.1, 129.6) | 60.5 (45.5, 87.5) | 2.472 | 0.014 |
| Anti-CCP, U/mL | 81.0 (70.6, 137.9) | 85.8 (56.5, 91.0) | 1.326 | 0.188 |
| hs-CRP, mg/L | 32.6 (31.9, 33.8) | 13.3 (12.7, 13.8) | 5.759 | <0.001 |
| ESR, mm/h | 57.5 (55.2, 59.1) | 28.0 (25.7, 29.6) | 5.759 | <0.001 |
| DAS28-ESR | 6.2 (6.1, 6.3) | 4.3 (4.2, 5.0) | 5.494 | <0.001 |

Patients with discordant RF and anti-CCP titers (one above and the other below the respective median). Statistical methods were the same as those for Table 1; Fisher exact test was used when the expected frequency was <5.

**Supplementary Table S4. Interaction analyses of TRAb with Ln(RF) and Ln(anti-CCP) (exploratory analysis, N=192)**

| **Variable** | **Model A**  **with TRAb×Ln(RF)** | **Model B**  **with TRAb×Ln(anti-CCP)** |
| --- | --- | --- |
| TRAb-positive | B=-1.739, P=0.281 | B=-4.632, P=0.015 |
| Ln(RF) | B=0.246, P=0.218 | B=0.480, P=0.008 |
| Ln(anti-CCP) | B=0.524, P=0.010 | B=0.191, P=0.368 |
| TSH, mIU/L | B=-0.090, P=0.050 | B=-0.083, P=0.067 |
| Age, years | B=-0.004, P=0.642 | B=-0.002, P=0.823 |
| Disease duration, years | B=-0.011, P=0.680 | B=-0.019, P=0.481 |
| BMI, kg/m² | B=0.330, P<0.001 | B=0.323, P<0.001 |
| Glucocorticoid use | B=0.239, P=0.151 | B=0.280, P=0.086 |
| TRAb × Ln(RF) | B=0.746, 95% CI (0.018, 1.475), P=0.046 | — |
| TRAb × Ln(anti-CCP) | — | B=1.360, 95% CI (0.538, 2.182), P=0.0014 |
| Model summary | *R*²=0.586, adjusted *R*²=0.566, *F*(9,182)=28.6, *P*<0.001 | *R*²=0.600, adjusted *R*²=0.580, *F*(9,182)=30.3, *P*<0.001 |

Interaction terms were added separately to the primary analysis model in all 192 patients. The TRAb×Ln(anti-CCP) interaction was significant (*P*=0.0014) and the TRAb×Ln(RF) interaction was marginally significant (*P*=0.046), suggesting that the association between TRAb and DAS28-ESR strengthened with increasing rheumatoid autoantibody titers. The main effect of TRAb in the interaction models represents the effect when the log-transformed titer equals 0 and should not be interpreted in isolation from the interaction term. Statistical interaction reflects the pattern of association strength varying across levels of another variable and does not constitute biological synergy or causal inference.
